# Supplementary material for: Brightly coloured tissues in limid bivalves chemically deter predators
Source: R Soc Open Sci. 2019 Oct 2;6(10):191298. doi: 10.1098/rsos.191298 (PMC6837197; doi:10.1098/rsos.191298)
Supplement: Supplementary method [file rsos191298supp1.docx]

**Brightly-Colored Tissues in Limid Bivalves Deter Predators and are Metabolically Unique**

Lindsey F. Dougherty, Alexandria K. Niebergall, Corey R. Broeckling, Kevin L. Schauer, and Jingchun Li

**1. Supplementary** **method**

*1.1. Specimen supply*

For the behavioral trails, disco clams (*Ctenoides ales*) and peacock mantis shrimps (*Odontodactylus scyllarus*) were purchased from Blue Zoo Aquatics (Hawthorne, CA USA). The Manila clams (*Venerupis philippinarum*) were purchased from Monterey Fish Market, Berkeley CA, USA. Collecting localities for the specimens are unknown.

For the metabolomics study, *Ctenoides* and *Spondylus* specimens were purchased from Aqua Imports (Boulder, CO USA). *Ctenoides ales* and *Spondylus sp.* were collected in the Philippines, and *C. scaber* was collected in Haiti. *Argopecten* were purchased from Farm-2-Market (San Francisco, CA USA) and collected in Boston, MA, USA. Specimens were deposited into the Museum of Natural History, University of Colorado Boulder (accession numbers 48125-48134, 48143).

*1.2. Ultra-high pressure liquid chromatography (UHPLC) coupled to time of flight (TOF) mass spectrometry (MS).*

From each specimen, four types of tissues (⋝5 mg dry weight) were extracted and frozen using liquid nitrogen: mantle, tentacle, gills, and adductor muscle. Samples were taken to the Proteomics and Metabolomics Facility at Colorado State University for analysis. Samples were lyophilized at 0.008 mBar, -80 °C, for 72 hours and weighed into 2 mL autosampler vials targeting between 5 mg and 15 mg. Samples that did not contain a minimum of 5 mg of dry tissue weight were pooled with samples in the same treatment group. 0.1 mL of MTBE/MeOH/Water

(6/3/1 v/v/v) was added per mg of dry tissue followed by vortexing at 4 °C for 1 hour. The samples were then sonicated in a QSonica Ultrasonic processor at 65% amplitude for a total of 10 minutes, 30 seconds on followed by a 30 second pause, before being vortexed for an additional 1.5 hours at 4 °C. 400 μl of water was added for each 1 mL of MTBE/MeOH/Water (6/3/1 v/v/v) and the biphasic mixture was vortexed for 30 minutes at 4 °C. The two phases were separated by centrifuging at 4 °C for 30 minutes at 3000 xg.

Samples were analyzed in discrete, randomized blocks with a pooled quality control (QC) injection after every 6 samples. The QC sample was generated by pooling a portion of all samples in the dataset. 150 μl of the lower aqueous layer was dried and resuspended in 100 μL of methanol/water (1/1 v/v). 150 μL of the upper organic layer was dried and resuspended in 100 μL of methanol/toluene (1/1 v/v). To ensure optimal metabolome coverage in a single run, a stacked injection approach was used (Broeckling and Prenni 2018). One μL of organic layer was pre-loaded onto a Waters Acquity UPLC system equipped with a Waters Acquity UPLC CSH Phenyl Hexyl column (1.7 μM, 1.0 x 100 mm) with a constant flow of 99.9% solvent A (2 mM ammonium hydroxide, 0.1% formic acid) and 0.1% solvent B (acetonitrile, 0.1% formic acid) at 200 μL/minute. This isocratic method was allowed to run for 30 seconds before the injection of 1 μL of aqueous layer. Separation was performed using a gradient that began at 100% A, was held at 100% A for 1 min, ramped to 98% B over 12 minutes, held at 98% B for 3 minutes, and then returned to starting conditions over 0.05 minutes and allowed to re-equilibrate for 3.95 minutes, with a 200 μL/min constant flow rate. The column and samples were held at 65 °C and 6 °C, respectively. The column eluent was infused into a Waters Xevo G2 Q-TOF mass spectrometer equipped with an electrospray source and operated in positive mode, scanning 50-2000 m/z at 0.2 seconds per scan, alternating between MS (6 V collision energy) and MSE mode (15-30 V ramp). Calibration was performed using sodium iodide with 1 ppm mass accuracy. The capillary voltage was held at 2200 V, source temp at 150 °C, and nitrogen desolvation temp at 350 °C with a flow rate of 800 L/hr.

*1.3. Metabolomics Data Analyses*

For each sample, raw data files were converted to .cdf format and matrix of molecular features as defined by retention time and mass (m/z) was generated using XCMS (v1.42.0) software in R (v3.1.1) (Smith et al. 2006) for feature detection and alignment. Raw peak areas were normalized to total ion signal in R. The “fillPeaks” function was utilized to remove zero values from the dataset. Thus, each molecular feature will have a value greater than zero, even if it was not detected in a sample. Outlier injections were detected based on total signal and PC1 of principle component analysis. Features were grouped based on a novel clustering tool, RAMClustR (Broeckling et al. 2014), which groups features into spectra based coelution and covariance across the full dataset, whereby spectra are used to determine the identity of observed compounds in the experiment. Compounds were annotated based on spectral matching (Tsugawa et al. 2016, Jarger et al. 2017) to metabolite databases [in-house, 1-SToP (Broeckling et al. 2016), NISTv14, Golm, Metlin, Massbank]. The peak areas for each feature in a spectrum were condensed via the weighted mean of all features in a spectrum into a single value for each compound. In addition, all compounds with a charge state greater than 1 are labeled as ‘peptides’.

A pairwise t-test was conducted on each compound using the “t.test” function in R, and p-values were adjusted for false positives using the Bonferroni-Hochberg method in the p.adjust function in R.

PCA was conducted on mean-centered and pareto variance-scaled data using the pcaMethods package in R. The Pareto scaled data would reduce bias toward high abundance compounds. A hierarchical clustering analysis was conducted on chemical compositions of all tissue samples based on Euclidean distances among samples using the R function “heatmap.2”.

**References**

Broeckling CD, Afsar FA, Neumann S, Ben-Hur A, Prenni JE. RAMClust: a novel feature clustering method enables spectral-matching-based annotation for metabolomics data. Analytical chemistry. 2014 Jun 26;86(14):6812-7.

Broeckling CD, Ganna A, Layer M, Brown K, Sutton B, Ingelsson E, Peers G, Prenni JE. Enabling Efficient and Confident Annotation of LC− MS Metabolomics Data through MS1 Spectrum and Time Prediction. Analytical chemistry. 2016 Sep 8;88(18):9226-34.

Broeckling CD, Prenni JE. Stacked injections of biphasic extractions for improved metabolomic coverage and sample throughput. Analytical chemistry. 2018 Jan 2;90(2):1147-53.

Jaeger C, Méret M, Schmitt CA, Lisec J. Compound annotation in liquid chromatography/high‐resolution mass spectrometry based metabolomics: robust adduct ion determination as a prerequisite to structure prediction in electrospray ionization mass spectra. Rapid Communications in Mass Spectrometry. 2017 Aug 15;31(15):1261-6.

Smith CA, Want EJ, O'Maille G, Abagyan R, Siuzdak G. XCMS: processing mass spectrometry data for metabolite profiling using nonlinear peak alignment, matching, and identification. Analytical chemistry. 2006 Feb 1;78(3):779-87.

Tsugawa H, Kind T, Nakabayashi R, Yukihira D, Tanaka W, Cajka T, Saito K, Fiehn O, Arita M. Hydrogen rearrangement rules: computational MS/MS fragmentation and structure elucidation using MS-FINDER software. Analytical chemistry. 2016 Aug 4;88(16):7946-58.
